# Complete plastid genome of *Holcoglossum tsii* (Orchidaceae, Aeridinae) and phylogenetic analysis

**DOI:** 10.1080/23802359.2019.1700838

**Published:** 2019-12-18

**Authors:** Yong-Bin Chen, Ming Xie, Sheng-Ting Guo, Xiao-Yong Zheng, Heng Li

**Affiliations:** Fujian Vocational College of Bioengineering, Fuzhou, China

**Keywords:** *Holcoglossum tsii*, plastid genome, phylogenetic analysis

## Abstract

The complete plastid genome of *Holcoglossum tsii* was determined and analyzed in this work. The plastome was 146,897 bp in length with 83,366 bp of the large single-copy (LSC) region, 11,957 bp of the small single-copy (SSC) region, and 25,787 bp of the invert repeats (IR) regions. The genome contained 127 genes, 74 protein-coding genes, 38 tRNA genes, and 8 rRNA genes. Phylogenetic analysis suggested *H. tsii* is sister to *H. rupestre*.

*Holcoglossum tsii*, described in Yunnan Province of China by Yukawa (2000), is a controversial species. The author stated that this species is readily distinguished *H. rupestre* by having shorter leaves, a smaller number of flowers, entire margins of sepals, a two-fold wide, mid-lobe of the labellum, and a two-fold long spur. Jin and Wood (2009) transferred it as a synonym of *H. rupestre*. Based on the nrITS, *matK* and *trnL-F*, the phylogenetic analyses showed that *H. tsii* is sister to *H. lingulatum* plus *H. omeiense* with weak support (Liu et al. 2011). However, after the addition of *psbA-trnH*, *atpI-atpH* and *trnS-trnfM* markers, the phylogenetic analyses showed *H. tsii* is sister to *H. quasipinifolium* and unstable topologies were recovered between the maximum likelihood, Bayesian inference and maximum parsimony (Zhang et al. 2013). Present study assembled the plastid genome and analyzed the phylogenetic position of *H. tsii*.

Fresh leaf sample of *H. tsii* was acquired from Luquan Yi and Miao Autonomous County (25°33′N, 102°28′E), Kunming City, Yunnan Province of China, and voucher specimen deposited at The Orchid Conservation and Research Center of Shenzhen, Guangdong Province of China (specimen code Z.J. Liu 9781). DNA was extracted from fresh leaf tissue, with 400 bp randomly interrupted by the Covaris ultrasonic breaker for library construction. The constructed library was sequenced PE150 by Illumina Hiseq 4000 platform, approximately 20 GB data were generated. Illumina data were filtered by script in the cluster (default parameter: -L 5, -p 0.5, -N 0.1). Paired reads were removed when N content in sequencing reads exceeded 10% of the read base number and the low-quality (Q ≤ 5) base number in sequencing reads exceeded 50% of the read base number (Yan et al. 2013). Complete plastid genome of *Gastrochilus fuscopunctatus* was used as as reference, the paired-end reads were filtered with GetOrganelle pipe-line (https://github.com/Kinggerm/GetOrganelle) to get plastid-like reads, then the filtered reads were assembled by SPAdes version 3.10 (Bankevich et al. 2012), the final “fastg” were filtered by the script of GetOrganelle to get pure plastid contigs, and the filtered De Brujin graphs were viewed and edited by Bandage (Wick et al. 2015). Then, we can get the circle plastomes. Assembled plastid genome annotation was based on comparison with *G. fuscopunctatus* by GENEIOUS v11.1.5 (Biomatters Ltd., Auckland, New Zealand) (Kearse et al. 2012). The annotation result was draw with the online tool OGDRAW (http://ogdraw.mpimp-golm.mpg.de/) (Lohse et al. 2013). The phylogeny based on the complete plastid genome shared by *Holcoglossum* species was inferred from the ML search and ML bootstrap analysis using RAxML (Stamatakis 2014); 14 representative species of Aeridinae were aligned using MAFFT v7.307 (Katoh and Standley 2013); bootstrap probability values were calculated from 1000 replicates and *G. fuscopunctatus* (KX871233) and *Phalaenopsis equestris* (JF719062) served as the outgroup.

The complete plastid genome sequence of *H. tsii* (GenBank accession MK836106) was 146,897 bp in length, with a large single-copy (LSC) region of 833,66 bp, a small single-copy (SSC) region of 119,57 bp, and a pair of inverted repeats (IR) regions of 257,87 bp (Figure 1). The complete genome GC content was 36.70% (LSC, 33.95%; SSC, 28.28%; IR, 43.12%) and the plastome contain 127 genes, 74 protein-coding genes, 38 tRNA genes, and 8 rRNA genes. The ML tree showed that the *H. tsii* is sister to *H. rupestre* with strong support.

**Figure 1.**
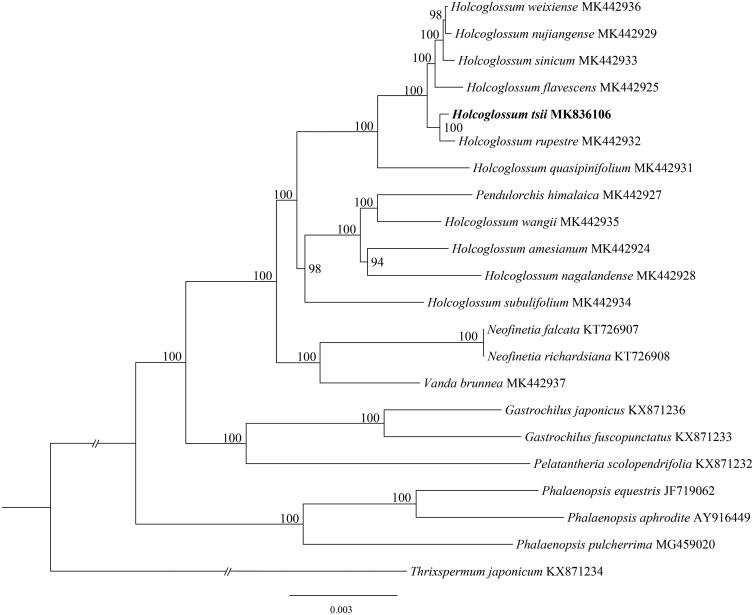
The maximum-likelihood (ML) tree based on the 22 representative plastid genomes of the subtribe Aeridinae. Numbers near the nodes mean bootstrap support value.
